# Ovariotomy

**Published:** 1859-08

**Authors:** 


### OVARIOTOMY.

At the time of writing a notice of this operation, we were not in the possession of the essay of Dr. Lymen, of Mass., published in 1856. This essay is in many respects the most complete which has yet been published on the subject, and contains some very just criticisms on tables and cases formerly published. It is deficient at the present date, in what regards the use of iodine injections for ovarian cysts. It appears that Dr. Lyman is preparing a new and larger edition of his valuable essay in which this defect will no doubt be remedied.

We notice in the last number of the *St. Louis Medical Journal*, a fifth operation by Professor Pope mentioned. The patient died one or two hours after the operation, and the tumor was found to be of a cancerous nature.

---

Operations for the radical cure of hernia are the prevailing mania of the profession just now, and instruments of various kinds are offered in all instrument makers' shops, by which the *manuel operatoire* may readily be effected.

This mania is a disease which only attacks a surgeon once, and, having experienced it several years since, we are at present exempt. We would say, therefore, to our friends, be not in a hurry to perform operations for the radical cure of hernia.

The operations now proposed, are essentially that of Gertly, in vogue about twenty years ago. If the effects are slight, it fails of success; if severe, it is dangerous.

